# Indigenous migrant labourers and land: towards an exploration of indigenous’ socio-cultural reproduction in the Colombian *Altillanura*

**DOI:** 10.1007/s10460-024-10693-x

**Published:** 2025-01-17

**Authors:** Lorenza Arango

**Affiliations:** International Institute of Social Studies (ISS) of Erasmus University Rotterdam, The Hague, The Netherlands

**Keywords:** Indigenous peoples, Migrant labor, Social reproduction, Land, Colombia

## Abstract

Production and social reproduction are increasingly characterized by its *multisitedeness* given the rising number of working people migrating for wage work. The ways in which these spheres interplay, both at migrants’ places of origin and at the destination sites of work, differ greatly across societies. Based on primary research in Puerto Gaitán, Colombia—a small town in a largely rural area serving as an agribusiness and oil exploitation hub—I suggest that the complex expressions of the production-social reproduction nexus are intimately connected with the ‘politics of land’. While the town of Puerto Gaitán, as a place of migration, provides for most everyday ‘material’ elements to sustain life (food, shelter, clothing, care, schools, hospitals), other crucial aspects of social reproduction are still heavily performed at indigenous’ rural collective lands (*resguardos)*. *Resguardo* lands are a key socio-cultural basis of Indigenous peoples’ reproduction, despite serious limitations partly due to their isolation, to restrictions on the free circulation by large landowners, and to historical underlying drivers founded in dispossession. One important analytical and political implication is that migrant workers’ access to land should respond to a broader conceptualization of social reproduction—i.e., in which non-material aspects are equally considered.

## Introduction

Across the globe, Indigenous peoples are increasingly migrating to urban and peri-urban areas in search of better livelihood sources (Arriagada et al. 2020; Finegan 2021; Guevara Ordoñez 2017; Horn 2018; ʻĀina of Kaʻōnohi et al. 2023). Within Colombia, a rise in the rural to urban migration of ethnic communities (i.e., indigenous groups and black ‘Afro’) is also a noticeable trend (Hernández and Franco 2021; Muñoz 2018; Nuñez Basante 2020).

In the case of the indigenous, the majority are based in collective lands (*resguardos*), certified by law, which are nevertheless unable to sustain life (Borda and Mejía 2006; Mosquera 2018; Sánchez 2019). Across the country’s *Altillanura* (eastern high plains), in particular, many such *resguardos* lack productive capacity (poor soil quality, insufficient hunting ground, destroyed oases, restricted access to water sources, and so on), basic infrastructure (e.g., roads) and services (health clinics, schools)—an aggregate result of centuries of land grabbing, genocide and discrimination They are located in remote, isolated areas, sometimes seven or ten hours away from the nearest town center. Traditional life-affording activities like hunting and foraging have become extremely difficult to practice, partly as a result of restrictions on the free circulation imposed by private landowners and companies—who are increasingly claiming control over lands surrounding the *resguardos*, or even inside them (see e.g. Arias 2024; Calle 2017a; Indepaz 2015; Ñañez Ortiz and Calle 2022; Rutas del Conflicto 2021a). Some indigenous communities have attempted to engage in agriculture and to sell their little produce, but the majority of the lands they are left to use are either too small for wildlife to thrive or water deficient, or a combination of both (see, e.g. Buitrago Escobar 2017). Still, even when indigenous are able to gather or generate a decent marketable surplus, they often face many other challenges in order to bring it to the local markets, such as the absence of roads and means of transportation. There are cases where *resguardos* are completely encircled by private landowners (*finqueros*) that they do not even have any right of way. Several of those who live in the *resguardos* are partly dependent on food aid from government and NGO programs. Under these conditions, households in these *resguardos* face recurring crises of social reproduction (Fraser 2016; Katz 2001; Mezzadri et al. 2024; Ojeda 2021), resulting in many being forced to leave and seek possibilities for life elsewhere (Ardila 2023; Martínez 2020).

And yet, for many of the indigenous migrant workers resettled in other, more urban-type of places (in my area of research in the eastern plains, these are mainly small towns in a largely rural region), *resguardos* are not a thing of the past. In fact, they work hard, among others, to be able to retain close ties with the rural lands they consider as being a part of. Some pay regular visits to their *resguardos* after each seasonal job finishes or in the period between one working shift and another. Others, unable to commute themselves, usually allocate a portion of the salary from their wage work to compensate family members and neighbors for looking after the houses, the food gardens (*conucos*) and the animals that they were forced to leave behind. Children and elderly (many of whom have relocated together with the indigenous adult workers in order to access education and health services, respectively) are usually sent back to the indigenous *resguardos* during school break and after medical treatments. Overall, and as stated by Calle (2017b) and Ulloa (2012) in relation to the Sikuani Indigenous peoples in the savannas of Vichada, and to the Kogui peoples to the north of the country, respectively, today *resguardos* represent one of the most significant bases of indigenous’ identity—something they cannot afford to lose. This recognition, however, does not abstract from the broader historical processes of dispossession underlying the emergence of *resguardos* in Colombia and the *Altillanura* region, in particular—as discussed below.

Outside Colombia, this is not an isolated case either. Penelope Anthias’ substantive work on indigenous communities in the Bolivian Chaco, for instance, also exemplifies the complexities of the connections between territory and ethnicity. She argues that despite ‘ethnic territories’ can be considered as expressions of colonial legacy—and thus should not be treated as the ultimate ‘foundation for emancipation’, it is also true that these sites that have been ‘reappropriated’ and ‘reworked’ by Indigenous people in their own political struggles, and thus carry special significance to their life projects (Anthias and Hoffmann 2021:223). This is not to say, as argued by Anthias, that the indigenous population in this Bolivian region is inscribed to the more modern notion of a ‘collective and bounded territory’ (similar to the *resguardos* in the Colombian case). But given that radical transformations in land use and ownership have changed the indigenous population’s ‘imaginaries and aspirations’, it is also true that Indigenous peoples *sometimes* conceive collective land titling as a mechanism for better land access and as source of livelihoods (Anthias 2021:276).

This paper draws on the experiences of indigenous communities belonging to different *resguardos* across the Altillanura in Colombia, who have migrated for work to Puerto Gaitán—a strategic hub of agribusiness and oil commerce in the region. It is based on research undertaken as part of a wider study investigating the character of the recent land rush in Colombia and its relation to the politics of labor and social reproduction, among other aspects.^1^ In 2022 and the first quarter of 2023, this broader interest on the land rush took me to several municipalities of the Altillanura, a region that had exhibited a significant demand for land in the contemporary period—coinciding with the ‘global land rush’ (White et al. 2012; Wolford et al. 2013, 2024). During this time, I learned from an informal settlement in the peri-urban area of Puerto Gaitán accommodating an increasing number of indigenous seeking for wage work. A majority of them were, at the *same time*, members of formally constituted *resguardos* spread across the region. With the assistance of a local NGO, the Norman Pérez Bello Claretian organization, I conducted (37) semi-structured interviews to indigenous migrant workers, and several members of their households at the settlement, inquiring about their motivations for migrating to town, their employment conditions, as well as their relationship with land both at the peri-urban settlement in Puerto Gaitán and at their rural home sites. This last issue was also explored in more depth though the application of a household survey of 58 participants, which I administered together with the assistance of the NGO.

Building on indigenous migrant workers’ accounts, I argue that while the town of Puerto Gaitán—a small, more or less urban destination site of indigenous migrant labour—provides for most of the everyday ‘material’ elements to sustain life (including food, shelter, clothing, care, schools, hospitals) (see Pattenden 2018), other crucial components of their social reproduction as a group are still largely practices at indigenous rural lands under *resguardos*, their home sites. In essence, *resguardos* may be seen as an important site of indigenous peoples’ socio-cultural reproduction (Asher 2020; Ojeda 2021; Ossome 2022; Ulloa 2012). Based on this connection, this paper seeks to contribute to debates around the ‘use value’ and ‘exchange value’ of land for production and social reproduction, by suggesting that the socio-cultural basis that *resguardo* lands offers might well be one concrete expression of the use value function of land. This has important analytical and political implications for how we think about land access, which deserves more concerted empirical research.

The remaining of the paper is organized as follows. The next section situates this research within broader discussions about land and labor, and production and social reproduction and their interconnections, as well as introduces key analytical concepts that shaped the analysis. Section three provides basic contextual information about the town of Puerto Gaitán and presents a brief overview of the recent land rush in the region, to help the reader better understand reasons behind indigenous recent labor migration and their effects on social reproduction. These effects are later explored in sections four and five, respectively. The final section discusses implications of the increasing multi-sited character of production and social reproduction for social justice struggles, particularly around land access.

## Labour, land and social reproduction

Land (as a shorthand for different land-based resources i.e., water, soils, forests, wildlife) is central to everyday life. This is especially the case in agrarian societies where access to land represents the basis of different peoples’ livelihoods, including peasants and indigenous. Because of this interdependence, changes in land access will necessarily have implications on the ways rural communities procure their subsistence. For instance, people dispossessed from their lands may be forced into wage labor to meet social reproduction demands. They might get a job in nearby lands or they may need to migrate to other areas looking for employment. In any of these scenarios, however, there is a rupture with their ways of living. To theorize the connections between land, labor and social reproduction in relation to indigenous, I draw on four areas of research.

First, there is the literature on the role of land and labor as two fundamental production factors in capitalism. Marx’s seminal accounts on the emergence of capitalism offer a key point of departure. In his analysis, Marx noted how the transition from “feudal exploitation” to “capitalist exploitation” turned both land use and human labour are subjected to capitalist accumulation (1976:874). He also noted that the development of capitalism simultaneously produced “a relatively redundant working population” (1976:782). Looking at the situation in the contemporary era, the numbers of the so-called “relative surplus population” (RSP) have reached unprecedented levels. In reference to particular case studies, for instance, scholars have noted how the intensification of market-led development programs during the last three decades (1986–2014) was accompanied by increasing numbers of unemployment and job insecurity. An emphasis on labor-intensive industries implies that “employment creation is hardly a concern” (Habibi and Juliawan 2018:666). Similarly, as argued by Li (2010), the growing levels of the RSP have to do with “the predicament of people whose labour is not needed by the global capitalist system” (Li 2011:281), but whose land is.

Second, and in close connection to the above, is the growing body of research on the social reproduction of today’s “working people”. The work of different feminist and Marxists scholars (see: e.g., Bhattacharya 2017; Fraser 2016; Katz 2001) has investigated how the survival of the RSP has largely depended on their ‘ability’ to bear with the costs of their social reproduction. By “working people” (Shivji 2017), scholars refer to the ever-growing number of people that have been forced to secure their subsistence by working extra hours, under a lack of social security services, often combining different sources of income at the same time, and increasingly by self-employing themselves in the so-called “informal sector” (Bernstein 2006; Shivji 2017).

Within this literature, observers have emphasized the interdependence of production and social reproduction spaces, as well as its increasing multi-sited character due labor migration. In examining the rise of mechanized agriculture in early-twentieth century California, for instance, Mitchell (1996) details how the production of a ‘landscape’ of agricultural abundance in California was only possible because of the unbearable working and living conditions that agricultural workers were subjected to. A recent study by Shah and Lerche (2020) similarly reflects on the dyad of production and social reproduction. Surveying the conditions of domestic labour migration in India, they describe how the exploitation of capital takes place simultaneously “at the site of production as well as through labour’s social reproduction”, both at migrants’ destination and at their home towns. Most notably, as they note, what are thought to be associated to social reproduction activities, exclusively, are in fact productive at the same time. Thus, analyzing capitalist exploitation in the broader sense requires an appreciation of both production and social reproduction as a *continuum* (Bhattacharya 2017; Fraser 2016; Mitchell 1996; Ojeda 2021; Ossome 2021; Pattenden 2018; Shah and Lerche 2020).

Furthermore central to production and social reproduction is **land** (Mezzadri et al. 2024; Ojeda 2021; Ossome 2021).^2^. Traditionally, access to land has been rendered equivalent to ‘farmland’ as the quintessential site of production. However, in the wake of rising numbers of ‘working people’—necessitating land for both production and reproduction—farmland alone results insufficient^3^ Diana Ojeda, for instance, emphasizes “the centrality of both the material and social conditions of reproduction” and the important role that different types of land played in it, which include gardens, as well as “water wells, riverbanks and wildlands” (2021:86,89,94). In this way, land is thus not only a site to produce use and/or exchange value—to employ Marx’s (1976) terms—for the social reproduction of labour. Land is at the same time crucial to other purposes. Asher’s (2020:951) study of land struggles in Afro-Colombian communities explores precisely this matter. She shows how territorial claims “are not only struggles over material resources of land and livelihoods but also attempts to broaden the meanings of ethnicity, territory, and politics”. Overall, and as Levin et al. reminds us: “struggles over means of both production and social reproduction remain as important as ever (…) [and] land remains an important focus of such struggles” (2018:876).

Echoing the ideas in these two bodies of scholarly work, I discuss how indigenous communities across Colombia’s eastern plains have been forced to leave their lands and to engage in migrant wage labor—adding to the “working people”—which in turn alters the ways in which social reproduction is procured. This takes us to a third body of work that focuses on migration and the particular “mobile character” of many indigenous peoples. This literature shows how migrations, of indigenous and Afro communities, are at the basis of the “historical formation of Latin America” (Moya 2018). Sedentarization, which is thought by many as a natural evolutionary process, is instead the product of the often violent imposition of colonial powers upon mobile cultures (Katzer 2019) and of “disciplining discourses” (Quicke and Green 2018:656). Indeed, as noted by Whyte et al. (2019:325), “settler colonialism is itself a structure of domination that arranges institutions to undermine indigenous motion, mobility, and adaptation”. But while the “historicity of mobilities and migrations of indigenous peoples” is crucial, a more recent type of migration to cities and labor hubs is an important noticeable trend. The latter results from a combination of multiple factors including land dispossession, forced displacement and a resulting lack of viable economic alternatives in rural areas (Arriagada et al. 2020; Hernández and Franco 2021; Velasco-Ortiz 2022).

Finally, a fourth area of research, which critically examines the relationship between indigenous peoples and land, and the meanings and values attributed to indigenous’ territories (Bryan and Cruz 2022; Daigle 2019; Hunt 2014; TallBear 2019; Tuck et al. 2014), is also key to the present analysis. Indigenous scholarship has extensively documented the struggles for land by the indigenous, especially so against the backdrop of modern neoliberal conceptions of territory. As explained by Radcliffe (2012:359), the consideration of many indigenous lands as seemingly “empty and unproductive” has served as a justification to appropriate their territories, dispossessing them “from their own complex and varied socio-natures”. Some examples of this tendency are: the implementation of road infrastructural projects cutting right across indigenous lands (as in the case of IRSA in Guatemala) (Grandia 2013), or the subjugation of indigenous to modern governance rules via green grabbing and the “touristification” of both their peoples and places (Guilland and Ojeda 2012; Rocheleau 2015).

Latin American indigenous scholars, in particular, have rightly specify indigenous’ struggles to defend their territory. From the Brazilian Amazon, Ailton Krenak resists the idea of separating peoples from the land “where one knows how to live and desires to live in”. In the face of increasing land dispossession, he specially warns against the “risks of the rural exodus”, whereby indigenous forced to settle in peri-urban areas are “automatically turned to poverty” (Kon 2023). In Guatemala, as argued by Tzul Tzul (2019), “the vigour of the indigenous collective resistance” is founded in the centuries-long struggle to “govern their lands” in their own terms. With respect to rampant agrarian extractivism in Ecuador, Delmy Tania Cruz and others similarly emphasize on the historical character of indigenous ‘resistance, and the ways in which these take new forms in the contemporary era. A recent research in mining extraction suggests that “dispossession associated to extractive mega-projects have an effect in urban spaces, increasingly populated, and exhibiting an ever-increasing crisis to sustain life” (Cruz Hernández et al. 2020). Lisset Coba, for her part, synthetizes these ideas: she explains how the situation of marginal populations, like indigenous and Afro, are never only racial or ethnic. They are, at the same time, social, economic, political and environmental in character. The point is thus to address the social inequalities of these populations attending to an “intersectional approach” (Cielo and Coba 2018:170, 177)—and combined research on the material and socio-cultural dimensions of these inequalities may allow us to study it more thoroughly.

## A case study of Puerto Gaitán: a contextual overview

**Fig. 1:**
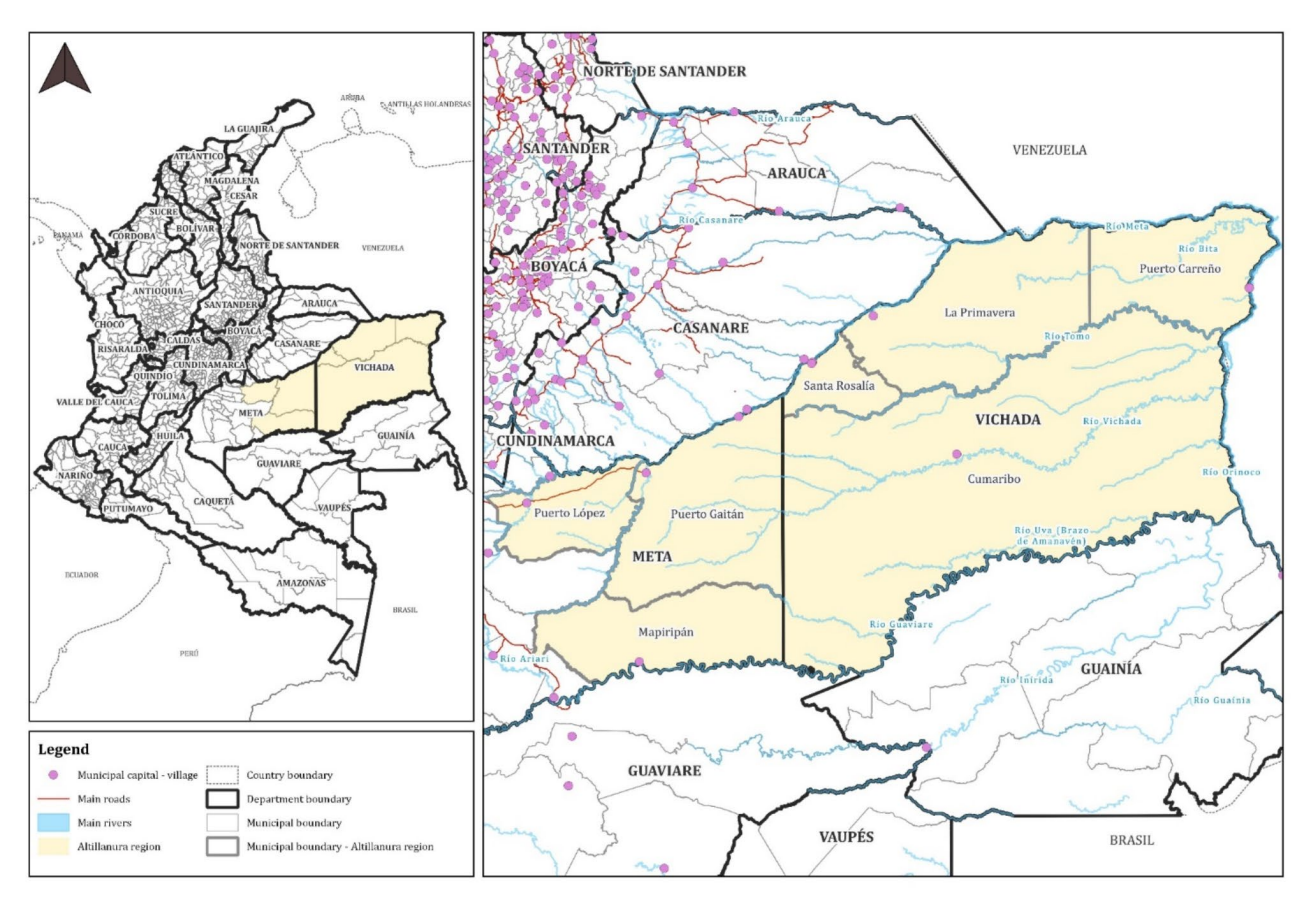
Map of the *Altillanura* subregion. Source: Created by Nicolás Rosero on behalf of author.

Puerto Gaitán is a municipality located in the department (akin to a province) of Meta, in the *Llanos Orientales* or eastern plains (also known as the Orinoco region) in Colombia. Although it is an area of relatively early ‘colonization’, with Catholic missions reported to be present from the mid-18th century, the first formal settlements date from the beginning of the twentieth century. According to historical accounts, one of these first settlements was founded in 1932 by a group of cattle ranchers, fishers and traders, along the banks of the Manacacías River—one of the main tributaries of Meta River, which connects various departments in the area and flows into the Orinoco, in the border with Venezuela (see Fig. 1). In fact, some of the first inhabitants of what today constitutes Puerto Gaitán originally came from Venezuela, most of whom were cattle-ranchers(Arias 2020:9–10). But for the most part, the vast savannas spreading across the country’s current Llanos Orientales were first inhabited by numerous nomadic indigenous communities (for example, Achaguas, Sálibas, Piapoco, Cuibas, Amorúa and Sikuani) that based their subsistence on slash-and-burn practices for food growing, and complemented their diets by fishing, hunting and foraging. Majority of these communities spent their lives travelling across the plains, by foot, and navigating the Orinoco and Meta Rivers, and some of its minor tributaries, on journeys whose duration could take weeks or months. According to some observers, their culture and traditions largely differed from some of the Indigenous peoples inhabiting other parts of Colombia, used to more sedentary practices (Millán 2021:13; Rausch 2013:2).

By early 19th century, towards the end of the colonial order initiated in 1500s, most of the Llanos’ territory had been looted. Their inhabitants, for its part, had been violently repressed and/or left with their identity fragmented by the action of Catholic missions. As noted by Baquero (1981), perhaps one of the few areas in which the indigenous managed to resist the Spanish invasion corresponds to the current territory of Vichada. Over time, due to its distance from the *piedemonte* (portions of the Llanos near the mountains), this became a strategic location for most Indigenous peoples of the plains, particularly for the Sikuani—a number of whose descendants are still based in the department today.

Still, a new wave of dispossession and repression would come with the wars of Independence during the first decades of the 19th century, in which the Llanos appeared as one of the key locations of the confrontation, and many Indigenous people were recruited to join the fight. In addition, the mestizo population increasingly migrated to the plains fleeing war in the Andes, and as the population of new settlers (*colonos*) expanded in the Llanos, the disputes over land also increased. Upon their arrival, several *colonos* joined in the extraction of different raw materials in the area such as rubber and quina, as the demands of international markets grew, which ultimately led to further encroachment on indigenous lands. On most occasions, the takeover of indigenous territories followed the perception of the latter as being ‘irrational’, ‘savage’ and ‘aggressive’ (Gómez 1991).

Once the republic was instituted in the mid-1800s, Indigenous peoples and their territories continued to be yet again subjects of constant abuse. The recently appointed government, led by Colombian leaders, labelled the Llanos Orientales as *baldíos* or state lands (Arias 2024) and as being of great economic importance but were presumed incapable of self-governance due to the ‘savage’ tribes (or *salvajes*, a derogatory reference to Indigenous peoples across Colombia) that inhabited the territory. In order to retain control over the frontier, they asserted the authority of Catholic missions (who had been long present in the area) over the Llanos and encouraged its further conversion to Christianity (Rausch 1993:25–41, 2013:2–3).

As of the late 19th century, the intendancy of Meta and the comisaría of Vichada, belonging to the Llanos Orientales, constituted two of various so-called ‘national territories’ (*territories nacionales*) in the country’s lowlands, following a new land classification introduced under the Republic that referred to areas located far from Bogotá city and with low population density (Serje 2011)—simply another way to name ‘frontier’ areas. At the time, the government also encouraged further colonization of the Llanos and pledged to improve road infrastructure and health services. Above all, however, it attracted *colonos* ‘through grants of free land’(Rausch 1993:104), further leading to the exacerbation of land disputes. In this regard, and as noted by González-Gómez (2015), it is worth noting that the territorial configuration of the eastern plains largely responded to discursive representations of the area (as in the references to it being a savage and frontier land), in addition to the more evident historic events and socio-territorial processes of colonization.

But it were not only Catholic missions that controlled the Llanos Orientales. The growing population of *colonos* also increasingly contributed to the transformation of the area and its ecology, a phenomenon that became most notable throughout the 20th century. To the segment of *colonos* that joined the extraction of raw materials, another group was added who later became large landholders, owners of big cattle ranches, both of whom continued to exert power over indigenous populations and their lands (Arias 2024:188–202; Gómez 1998; Martínez de Castellanos 1979; Serje 2011). Throughout the 1900s, migration in the Llanos was both driven by violence (in the form of different wars) and forced displacement in the highlands, and it was also the product of the state’s agenda, as the central government continued to issue *baldío* lands in the lowlands to encourage new settlements (Aragón 1983; Arias 2024; LeGrand 1986).

As of the mid-1900s, another period of war would leave the region at the mercy of self-defence peasant guerrilla groups and their counterparts (that is, the army, and armed factions of the Conservative party), which complicated further the dispute around access and control over land. Many Indigenous peoples were used as instruments of warfare by both contending groups, and several ended up abandoning the region or had no other choice than to advance further east. Pressures also came from yet new waves of migration, this time fleeing the hostility of *La Violencia* (a period of generalized violence in Colombia (often termed as ‘La Violencia’) (Fajardo 2015), and largely characterized by intense struggles over land access between the landed elites and campesinos *colonos* or peasant settlers), which turned the Llanos into one of the main destinations of peasant colonization (La Rota-Aguilera and Salcedo 2015:80) Very often new *colonos* took control over indigenous lands founded on the same principle that had justified earlier periods of usurpation: their alleged condition of ‘savages’ (Vargas 2005), and perpetrated dreadful massacres until well into the late 1960s, according to official reports (Gómez 1998). The infamous practice of the ‘indigenous hunts’ or *guajibiadas* became normalized by different strata of the population and newspaper reports of the time reproduced statements such as: ‘we didn’t know that killing indigenous was wrong’. Police, government officials, peasant *colonos* and large landowners alike were reported to partake in these massacres (Arias 2024:111–16; Millán 2021:105–18).

Against this background, the national government constituted the first indigenous reserves (*reservas indígenas*) in the region (Arias 2020:11, 2024), the recent antecedent of the indigenous *resguardos* (Gómez 1979; Mora Vera 2016; Rodríguez 2017) in the country’s post-colonial history (see Asher and Ramamurthy 2020; Gutiérrez-Escobar 2023).

To an extent, the *resguardos* have conferred indigenous with a relative protection of their lives, as well as access to land (see Ulloa 2012)—without disowning their shortcomings (e.g., confinement and an overall transformation of indigenous practices as a consequence of sedentarization) (Arias 2024:119–21; Calle 2017b). Several *resguardos* formally created across the eastern plains, mainly during the late 1980s and the 1990s. Notably, at the time, struggles for formal land recognition through establishment of *resguardos* were largely promoted by the largest indigenous organization in the eastern plains—which had been created in the previous decade and grouped different indigenous peoples in Meta and Vichada under the name of Unuma. In this sense, and as noted by Calle Alzate, state’s recognition of indigenous resguardos “can be seen as a demand of indigenous themselves”, in response to rampant land dispossession across the region (2023:220), as discussed above. In addition, in the Sikuani tradition, Unuma is also a word in the indigenous language that stands for ‘communal’, and ‘collective work’. Unuma is likewise the name of an informal indigenous settlement located in Puerto Gaitán which I argue illustrates the complex interplay between production and social reproduction today, and the role played by land politics in it, which we will explore in later sections.

The escalation of drug trafficking as of the 1980s, together with the recurrent peaks in paramilitary’s incursion from the late 1990s (Ballvé 2020; Grajales 2011, 2013; Gutiérrez Sanín and Vargas Reina 2016; Gutiérrez-Sanín et al. 2023; Peña-Huertas et al. 2017), led to new waves of violence and expulsion throughout the region (CNMH 2018:60). Still, notwithstanding this conflict-scenario, starting in the early 2000s the economy of Puerto Gaitán was on the rise, largely associated to an oil boom. In terms of population alone, commentators suggest that the transformation of the municipality was notorious: it moved from circa 18,000 inhabitants in 2001, to a little over 22,000 in 2007 and to some 30,000 by 2011, apart from the floating population of some 6000 people looking for wage work in the oil industry around the time. By the mid-2000s, the increasing dynamism of Puerto Gaitán was also attracting foreign investors. In 2008, a Canadian company acquired use-rights of an old oil well and turned it into the largest oil extraction site of the country to these days (Rivera Huertas 2023:237–39). At the same time, the ascent of Puerto Gaitán’s economy during this period can be attributed to a recent land rush in the *Altillanura*, a sub-region of the eastern lowlands of which Puerto Gaitán is part.^4^

Indeed, a renewed interest in land in Puerto Gaitán became more noticeable as of 2008 when the municipality turned the setting of an annual forum discussing the riches and (productive) possibilities of the *Altillanura* region. In 2008, together with one of his closest collaborators, the recently appointed mayor of Puerto Gaitán conceived the idea of the regional forum. As the mayor’s collaborator explained to me, while the mayor had always dreamed with the prospects of the region as an agro-industrial power, he saw his administration as a great opportunity to realize these aspirations. The mayor’s dreams found resonance with national-level discussions at the time that were repeatedly speaking of the *Altillanura* as the “last agricultural frontier” of the country (see Semana 2007, 2010a, b).

Both the local and national-level praise for the *Altillanura* (see Arias 2024:392–421; Díaz 2016 for a critique) and its seeming productive potential (see Ministry of Agriculture 2004), in turn, are best understood as one expression of a ‘global land rush’ (Wolford et al. 2024) that roughly took place between 2007 and 2018, with specific variations according to each country or region (e.g. Latin America (see Ojeda 2018); Europe (see van der Ploeg et al. 2015); Southeast Asia (see Peluso and Lund 2011; Schoenberger et al. 2017). A convergence of multiple global crises (food and oil prices, financial) triggered different investors to target lands across the globe for varied purposes like biofuels, large-scale agriculture and urban real estate development (see Cotula 2012; Fairbairn 2020; Neef et al. 2023; White et al. 2012; Wolford et al. 2013; Zoomers 2010). Here I understand the land *rush* as a particular conjuncture resulting in a rapid and unprecedented scramble for land, as defined by Borras and Franco (2024).^5^

As suggested above, Puerto Gaitán was already a dynamic boom town, largely in response to oil extraction in the area. Yet on the occasion of the different forums, the municipality grew exponentially; new and luxurious hotels, restaurants, clubs and resorts opened up to accommodate the flurry of attendees. New investments in land consolidated in the years that followed (2008 onwards), while some of the existing ones took advantage of the increasing interest for land and also expanded their operations (Ñáñez Ortiz and Calle 2017). To date, Puerto Gaitán houses the largest rubber plantation in Colombia (Las2orillas 2023). It is also the site of a leading meat-processing company country-wide, which also grows soy and maize for pig feeding (Portafolio 2008, 2023). Similarly, large-scale oil palm cultivation has experienced a major expansion in town (Semana 2019). More recently, Puerto Gaitán has also been at the center of public debate as the setting of one of the soundest land grabs in the context of the recent land rush in the area, in which a community popularly referred to in town as “The Menonnites” has taken control of over 30,000 hectares of land (Daniels 2021; Fitzgerald 2023; Rutas 2021b).

Puerto Gaitán is likewise the location of various middle-size private individual land plots or *fincas* mainly destined to rubber, oil palm cultivation and cattle ranching. Several of these *fincas* are owned by former politicians and leading business people from within the region, as well as from other country areas or from abroad. A number of these private *fincas*, in turn, were consolidated as a result of previous waves of land dispossession in Puerto Gaitán (Arias 2024; Ramírez and Ortega 2020; Rodríguez González 2014), and many benefitted too from the latest land rush. As the land rush facilitated yet a further encroachment of indigenous lands, these same indigenous were forced to look for alternative livelihoods elsewhere, including in urban Puerto Gaitán. The above contextual background is thus key for understanding indigenous’ most recent migratory trajectories. Here I invite a distinction between the historical practices of nomadism and territoriality of the indigenous across Colombia’s eastern plains, and the rural to urban migration taking place today—which I argue is largely motivated by contemporary land grabbing and the associated search for wage work, as explored below.

## Life at *Unuma* and the satisfaction of everyday (material) needs

As hinted at in the introduction section, several of the *resguardos* across the Orinoco savannas in Colombia are located in remote, isolated areas with poor or non-existent basic infrastructure and services—resulting from centuries of multidimensional violence described before. They lack of electricity and drinkable water, are poorly connected by road, and do not have access to nearby public health clinics, schools or local markets. Although some *resguardos*, especially those with the largest area, have portions of land destined to schools, they often cannot afford to hire a permanent teacher nor do they have basic school equipment like chairs and desks—making it impossible to secure access to education in practice (personal interviews, 06/2022).

On top of this, indigenous *resguardos* are increasingly being surrounded by private *fincas*, and by agro-industrial, mining and oil complexes—which have restricted access by indigenous to different types of lands, such as those close to rivers and spring waters, forests and (otherwise communal) pathways. In particular, with the arrival of new landowners to the area—especially in the context of the recent land rush—many of indigenous’ work sites were subsumed under private property signs, which have prevented them from using the land as an important source of food and livelihoods. Buitrago’s ethnographic study of the Sikuani Indigenous peoples in general (that is, not specifically of the communities referenced here) helps explain this situation:

In this review of the geographical and of soil’s scientific character in the areas in which the Sikuani people have been found since pre-Hispanic times, which largely correspond to areas today occupied by *resguardos*, a variety of land uses is observed. Recognizing the difference between areas of the Orinoquia gives an indication of the problems that arise with changes in the rhythms of life brought with colonization and sedentarization. On the one hand, the so-called nomadic groups were not only such because of their “character and idiosyncrasy” as they used to describe them, as if it were a matter of stubbornness, but because the qualities of the terrain were not the most favorable for sedentarization, especially in the *Altillanura* (Buitrago Escobar 2017:55).

**Fig. 2:**
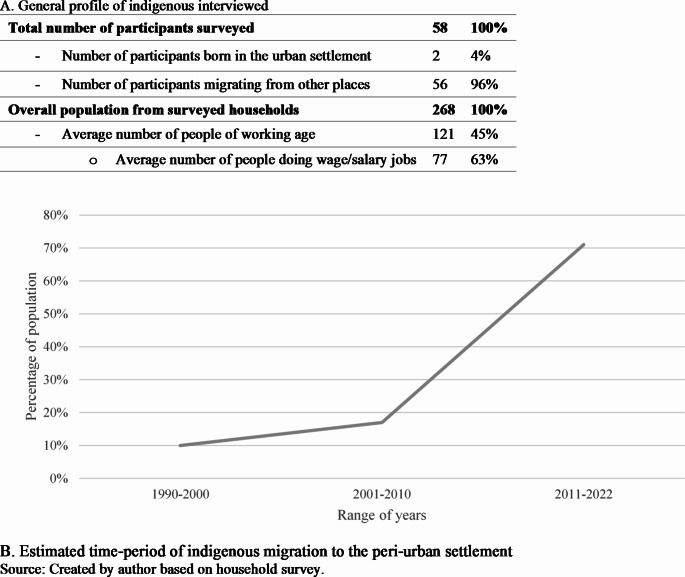
Indigenous labour migration to peri-urban Puerto Gaitán

As a consequence, over time indigenous peoples have had to migrate in search of better livelihood opportunities (see Martínez 2020; Rutas 2021b). The town of Puerto Gaitán, as noted above, has been a major recipient of different waves of indigenous migration. In the past two decades—coinciding with the renewed importance attributed to the *Altillanura*, of which Puerto Gaitán serves as an important hub—the number of indigenous migrating to town has significantly enlarged (see Fig. 2).

In Puerto Gaitán, indigenous are employed at oil palm and rubber plantations in the rural area of the municipality. They also work in the oil sector performing different roles such as security guards, machinery operators, cleaning staff and training instructors. Some serve as kitchen assistants, housemaids or construction workers in the urban area of town. Others, especially women and the elderly sell handicrafts (hats, purses and house ornaments) or traditional indigenous food such as *casabe* or *mañoco*—both of which are by-products of cassava, one of the main staple food crops in the region (see Table 1 for a stylized depiction). They must wait for long hours for customers, typically at the town’s main square or by the pier. One of the indigenous women I talked to described their situation in this way: ‘we look as if we were beggars, as it happens in the big cities’.

**Table 1:** Typology of wage jobs practiced by indigenous migrant labourers in Puerto Gaitán Source: Created by author based on household survey

Types of wage/salary jobs done by household members	Frequency	Percent
Seasonal farm work in other farms	26	33
Informal job	25	32
Formal job in other sectors	12	15
Formal job in the oil sector	11	14
Running small trade (cooked food, retailing, taxi, mototaxi etc.)	3	3
Total	77	100

Today, a vast majority of the indigenous migrant workers arriving in Puerto Gaitán are congregated in an informal settlement located right on the outskirts of town, popularly referred to as the village of Unuma or urban Unuma (‘Unuma *pueblo* or Unuma *urbano*’) (Unuma for short).^6^ Originally, as part of an agreement with the local government during the 1970s, a number of houses were built in Unuma to accommodate Indigenous peoples belonging to different *resguardos* from across the eastern plains. These were meant to serve as temporary foster homes that indigenous could make use of on different occasions, whenever they needed to commute to town—for example, for running errands, to attend medical appointments, to participate in special gatherings and assemblies, to transit in their trips to other destinations, and so on and so forth (personal interview, 07/2022). Houses were made of concrete and were relatively spacious with three or four large rooms and several bathrooms. Most are still in place to date, though in extremely poor condition due to a lack of maintenance. Each of these houses was assigned to a particular *resguardo*

Over time, however, Unuma ceased to be a transitory site and became the permanent place of residence for many indigenous migrating for work. At present, the informal indigenous settlement of Unuma comprises a little over seven hectares of land and is home to an estimated 79 families or 310 inhabitants. Some households living in the *resguardos* have relocated entirely to town, and so while adults perform different wage jobs, of the kind enumerated above, children are able to attend school, and elder members can also receive medical attention. Access to schooling and medical services are two of the most important aspects valued positively by indigenous resettled in Unuma. Paid jobs (formal and informal) available in Puerto Gaitán have also allowed indigenous to buy cleaning supplies and clothing that they could not afford in the past, and to acquire motorbikes for their daily commutes, to list just a few.

Still, despite that income from wage work in Puerto Gaitán certainly allows indigenous to cover important everyday needs, life in town is no less difficult. Indigenous I talked to complained about the challenges to survive in an increasingly populous, urban atmosphere that remains alien to their culture and values. For instance, they protest against high noise levels coming from loud music playing at bars and discos—many of which have opened up in recent years, even inside the settlement. Noise contamination is also due to the passing of heavy machinery going to oil camps and large-scale commercial plantations. Youth alcohol and drug abuse is another concern for many, as the demand for these among indigenous has increased exponentially, partly due to the presence of bars and other leisure places. During my field visits to Unuma, it became common to witness young kids, some ten or twelve years old, drinking beer; meanwhile, others, usually smaller, were playing with empty alcohol cans and bottles.

**Fig. 3:**
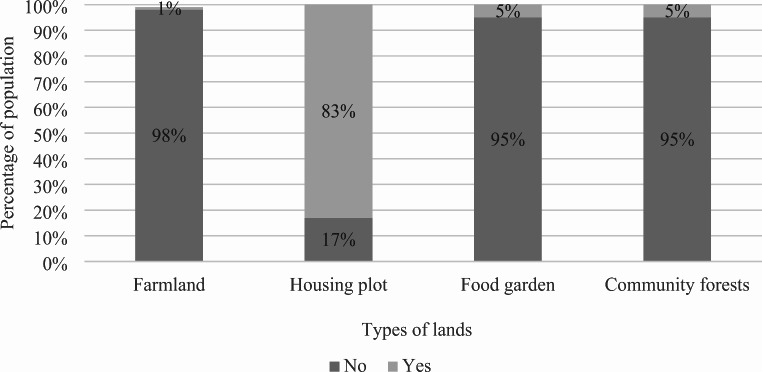
Access to land at Unuma (multiple choice answer). Source: Created by author based on household survey.

**Fig. 4:**
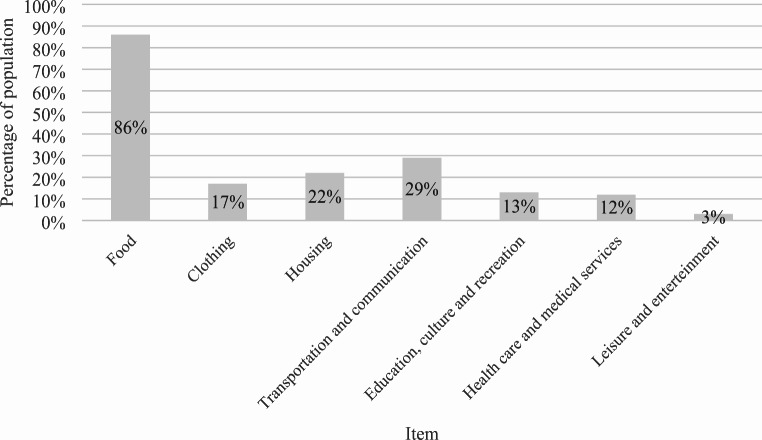
Household main expenses at Unuma (multiple choice answer). Source: Created by author based on household survey.

Apart from the few original houses made out of concrete, several living places in Unuma consist of improvised shelters covered by plastic sheets or by tiles of aluminium, in the best-case scenario. Sometimes one shelter is home to three households or more. Shelters are located right next to each other, with little or no space in between. Access to electricity and water is self-provided by the indigenous—by way of hanging off wires to the main electric cable, and by channeling water into secondary pipelines that are, in turn, directed to the different shelters. As the number of inhabitants increases, land availability at Unuma simultaneously reduces. There is no land to build more shelters, let alone for other vital purposes such as growing food or opening communal sites (see Figs. 3, 4). One of the indigenous people I talked to expressed: ‘When we arrived, we suffered, we endured hunger… We saved every cent to build the house. In my family, my husband is the one who works. He works on a farm outside of the town. He only comes on weekends’ (personal interview, 07/2022).

Some indigenous simply start building their shelters—and bring their family members along—without consultation with indigenous authorities of the settlement. This situation has made even more evident the need of more and better access to land in the urban area, and not only in the rural *resguardos*. As Darío*, one indigenous leader, described to me in our conversations, he no longer knows who is living in Unuma; his own informal census has been outdated for years, and even ‘white people’, non-indigenous are said to be living in the settlement. The latter are actually the owners of many of the bars and discos that have opened up inside the settlement in recent years; indigenous see an opportunity to proffer their tiny pieces of land in Unuma to the non-indigenous in Puerto Gaitán, which were rapidly emerging as key land brokers—he commented. This land shortage raises yet other concerns for Unuma’s inhabitants.

**Fig. 5:**
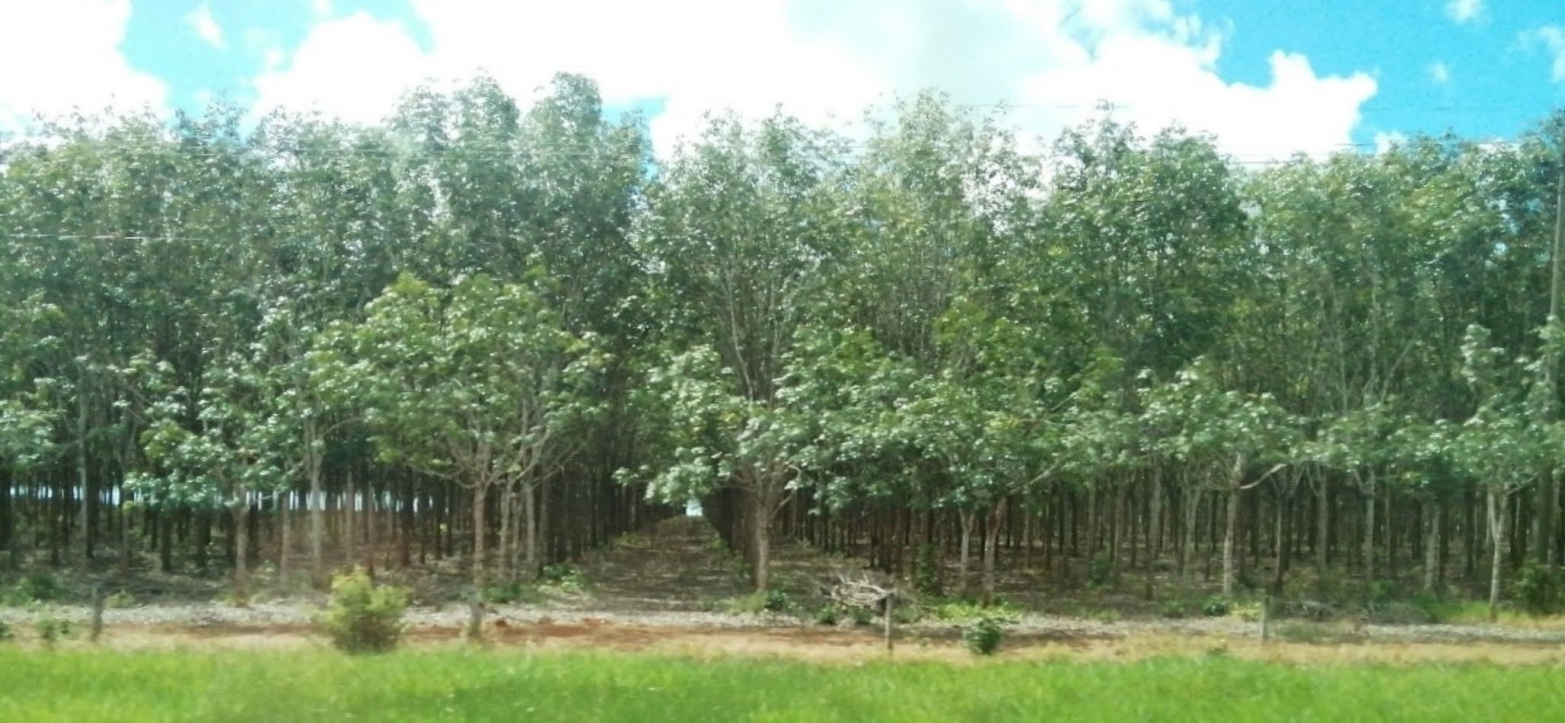
Rubber plantation belonging to the company Mavalle, along the main road from Villavicencio (the capital city of Meta) to Puerto Gaitán—some kilometers before the entrance to town. Photo by author, 2022

**Fig. 6:**
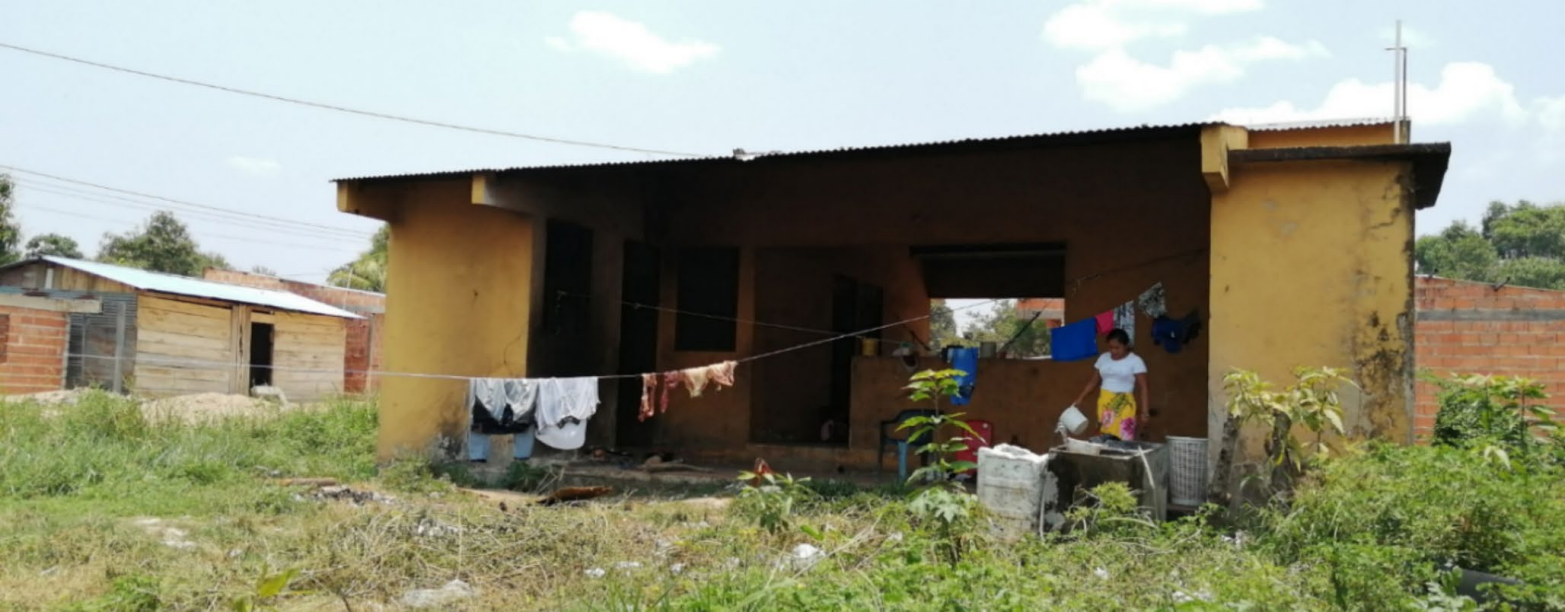
Informal settlement of Unuma on the outskirts of Puerto Gaitán, Meta. Photo by author, 2022

At the settlement, apart from the lack of drinkable water and electricity, there is no sewage system neither there is a proper service for garbage disposal. As one indigenous person described, several household members, especially the children, have become ill from consuming food and drinks contaminated with human waste. In a way, similar to Mitchell’s (1996) accounts on the California landscape, the production of the agribusiness and oil extraction landscape of Puerto Gaitán (Fig. 5) is the result of the hard work of hundreds of migrant workers, including the indigenous at Unuma, who are at the same time struggling for their own reproduction (see Fig. 6).

And yet, as indigenous explained to me, life at Puerto Gaitán is (only) slightly better than at the indigenous *resguardos* due to availability of paid jobs contributing to cover some of the costs of social reproduction. These, as we saw, cannot longer be met at their *resguardos* even if they are legally entitled to it, as a result of historical and contemporary land dispossession. Up to this point, Unuma as the destination site of migrant workers seems to concentrate both production and social reproduction activities, especially given that several household members have all relocated to Puerto Gaitán (including children and the elderly), and not only those engaged in migrant work. But as we shall see below, living in Unuma has not made Indigenous peoples less dependent upon their *resguardos* for other important reasons.

## *Resguardo* lands: elements of the socio-cultural reproduction of (indigenous) life

*Resguardos* continue to play a determining role in indigenous lives, notwithstanding their limitations (Calle 2023, 2024; Ulloa 2012). I see *resguardo* lands as one important expression of the socio-cultural reproduction of Indigenous peoples, in line with a wider interpretation of social reproduction as encompassing the material, as well as the cultural, political, social, economic and ecological aspects of reproduction (Bhattacharya 2017; Federici 2004; Fraser 2014; Katz 2001). Andland is central to these intersecting axes (Ossome 2021, 2022) (as a shorthand for different land-based resources, that is, water, soils, forests, wildlife, and as a homeland, territory and landscape altogether) as the place that represents the ‘possibilities to sustain life’ (Ojeda 2021:86). In fact, based on her study of indigenous and Afro-Colombian territorial struggles more generally, Kiran Asher considers ethnic territories as an ‘ethnic homeland’ (2020:965). Diana Ojeda, for her part, has extensively discussed the centrality of land to socio-cultural reproduction in reference to other rural settings in Colombia, but her ideas nevertheless resonate in variegated contexts such as the one we analyze here. A recent publication by Ojeda (2021:89), for example, stresses the significance of women’s access to land, and specifically to gardens, which “they saw as an important component of their campesina [peasant] identity”. The possibility to access different types of plants, as noted by the author, was also fundamental to other factors such as “to control their sexual and reproductive life using their knowledge of plants”. Both these arguments draw on and contribute to the growing academic literature on the interplay between land and social reproduction in Colombia.

In the *Altillanura*, we will see how *resguardos* also played into the social and cultural elements that sustain indigenous life and the ways in which these lands are central to their identity. Several of the indigenous migrant workers and their families resettled in Unuma still mantain a relation with the lands in which they were born. They try hard to keep their houses standing, as well as their food gardens (or conucos), even if nowadays they spend much of their time in town. A portion of the income from their wage work is often kept for house reparations at the *resguardos* and to grow the land, as well as for covering transportation expenses from the urban area of Puerto Gaitán. Some of the indigenous manage to travel to their *resguardos* in between working shifts, while others pay family members or neighbours to check on their land plots from time to time—in a way that keeps their practices of collective work (one of the different meanings of unuma) alive.^7^ During school vacations, many of the kids are also taken to the lands in the rural area to strengthen their ties with their original sites.

In addition, many indigenous inhabiting Unuma carry with them some of their agricultural produce from the *resguardo* on their way back to town. They miss the most some of the food preparations that are at the core of their diets such as mañoco and casabe. As noted above, besides directing their own produce to household consumption, some of these by-products are sometimes sold in Unuma to indigenous neighbours that cannot afford to commute to their *resguardos*, or who have lost access to their lands outright, and even to people outside the settlement—given that indigenous traditional food preparations have gained some recognition across the municipality. On occasion, indigenous also bring along palm fibre (moriche)—one of the most representative types of vegetation throughout the eastern plains—which they use for the making of some of the traditional handicrafts they sell in town.

**Fig. 7:**
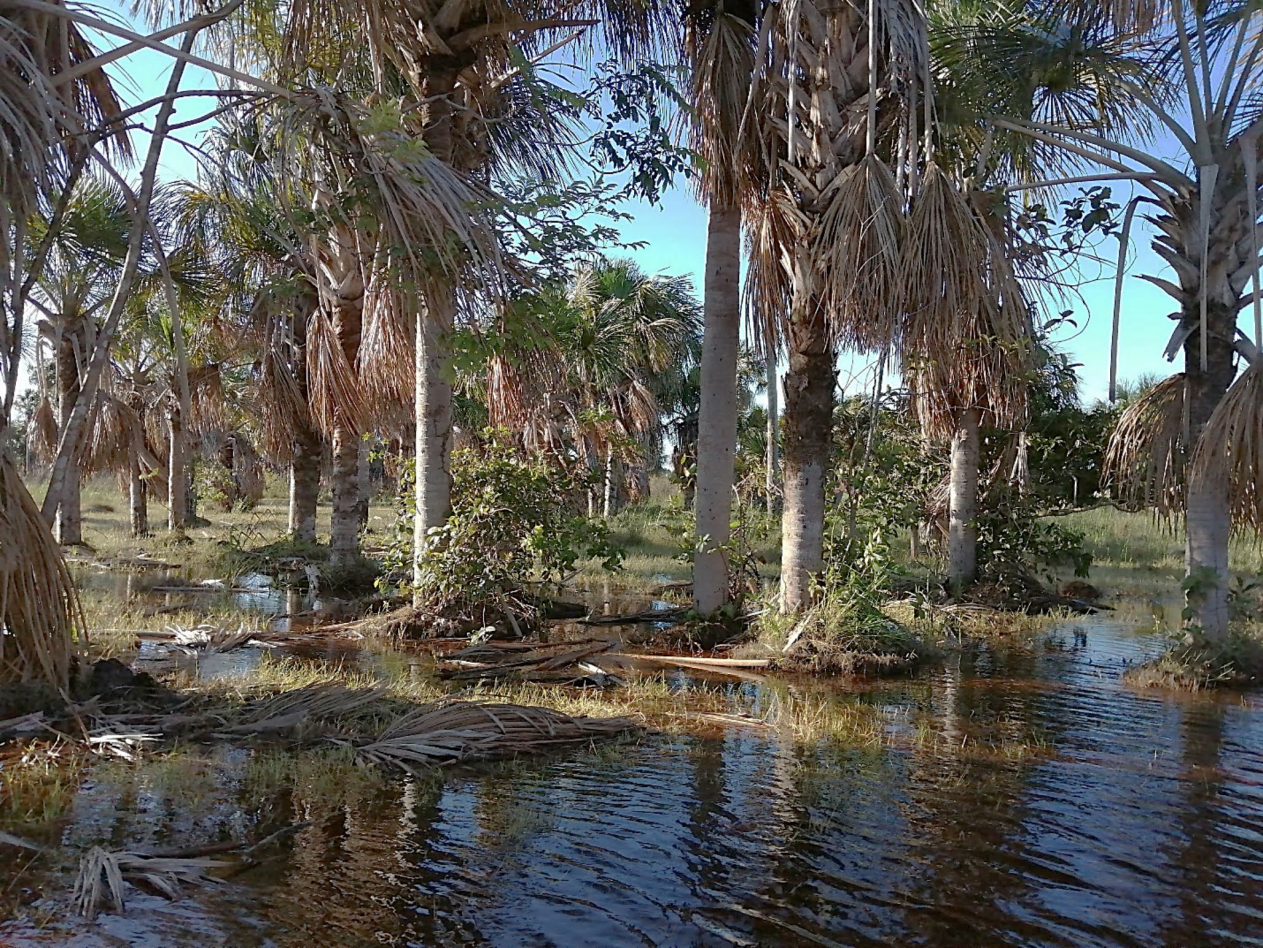
*Morichales* or moriche palm swamps at the indigenous *resguardo* of La Llanura in La Primavera, Vichada. Photo by the author, 2022

**Fig. 8:**
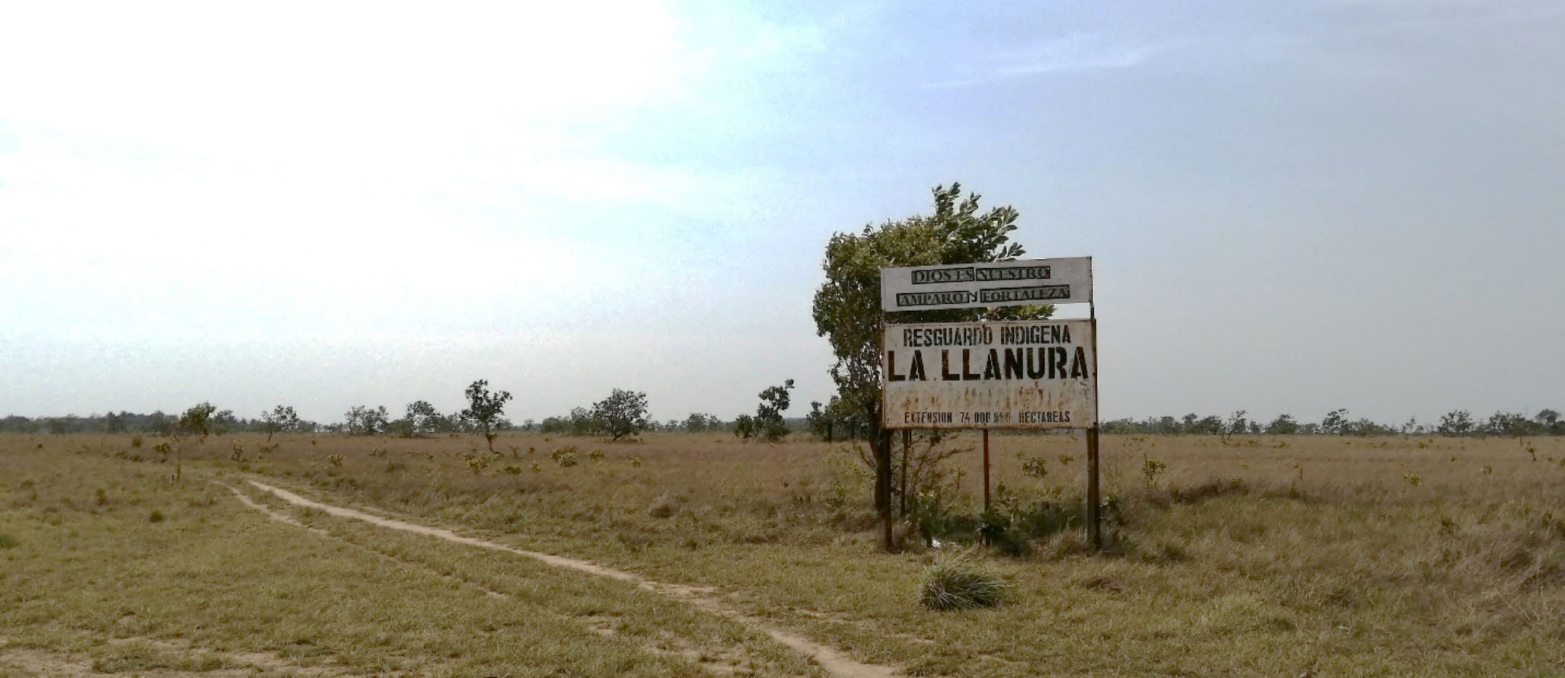
Entrance to the indigenous *resguardo* of La Llanura in La Primavera, Vichada. Photo by author, 2023

Despite better access to hospitals and medical care for the elderly (and other household members in general) being one key motivation to stay in Unuma, elder indigenous wish they could spend the final years of their lives on their ancestral lands. Some of the elderly people that I got the chance to talk to fear that they might die away from the lands they know and that they have considered their own. And so, as soon as their health condition improves, and provided that money is available to pay for the ticket, they are usually sent back to the *resguardos*. They ask to be buried next to their family members in places they refer to as sacred sites (Figs. 7, 8).

Other types of lands, such as moriche palm swamps (morichales), river banks and open savannas are also highly treasured by Indigenous peoples. They missed the times when they could enjoy better access to swamps and practice different activities such as to grow more and better food, and gather and forage things from the surrounding lands—which are typically more nutrient-rich due to higher water availability. Easy access to river banks is central for basic material reproduction such as cooking and cleaning, but also for the necessary leisure and socialization among community members. At Unuma, as one indigenous woman that I talked to lamented:

[…] here in town if you don’t work you don’t eat; there is no time to relax […]. My kids usually beg my husband to take them to the public swimming pool because they missed the times when we used to swim and play in the river, but he works at the oil camp and has no free time. Even when he is off, he is too tired to take them to swim; he just wants to sleep in (personal interview, 11/2022).

The feeling of freedom and the fresh air that one can enjoy in the open savannas is another crucial aspect that Indigenous peoples value of their original lands. One Indigenous person settled in Unuma described their current situation:

Coming from the *resguardo*, one is not used to so much noise. That causes stress, fatigue, and headaches, but one has to adapt. In the territory of the *resguardo* you live peacefully. Here in Unuma one lives confined, you no longer have the freedom to take a bath in the river or the freedom to go hunting (mariscar) in the savanna, which is our indigenous tradition (personal interview, 11/2022).

On the whole, those who strive to retain a strong connection with their *resguardos* do so as a means to obtain reassurance. The migratory trajectory of the indigenous population, going back and forth between their *resguardos* in the rural area and the settlement in peri-urban Puerto Gaitán, should not be conflated with their long tradition of nomadism—as hinted at above. I propose, instead, that this trajectory is seen as a tool against the commodification of everyday life (including their food ways) in the urban area, where they initially migrated to as part of the transformations in land use and ownership in the rural areas—largely exacerbated by the land rush. The work by Vélez-Torres (2013) in other parts of the country provides evidence that is useful to this proposition. She suggests that ‘the causes of the migration of Indigenous people to cities are varied and articulate violent productive and extractive processes with rural impoverishment and the socioeconomic marginalization of its inhabitants […]’. Moreover, she added:

These causes or motivations for displacement are not discernible in practice, since the presence of illegal armed actors is correlative to the selective and insufficient presence of the State, which is in turn related to the permissiveness of the State against the incursion of armed forces and illegal activities in rural areas […], especially when this territorial control favors private interests […]. The joint analysis of social marginalization, economic interests and military control, legal and illegal, evidence a historical constant of subalternization linked to the way in which the territories of ethnic communities have been spaces of intervention, exploitation and greed (2013:162).^8^

The transformation of indigenous territories resulting from marginalization and the pressures imposed by specific economic interests is indeed one of the motivations behind the most recent migration by indigenous populations to Puerto Gaitán. But as I have discussed above, despite the limitations of the *resguardos*, these are places of profound use value for Indigenous peoples to safeguard their traditions. And so, many are trying hard not to entirely disconnect from their *resguardos*. Additionally, consider other important aspects that can make Indigenous peoples cherish their *resguardos*. We saw that few of the indigenous migrant workers have a proper employment contract in town. The majority of the people I conversed with work as day labourers, at jobs of three to four months each and with long periods of unemployment in between. Furthermore, as the expansion of the municipality proceeds and new commodity and land booms arise, land appreciation increases, especially in the peri-urban area where Unuma is located. In consequence, threats of land dispossession in town are likely to become more prevalent as well. This uncertainty about their future in Unuma, and in urban Puerto Gaitán more generally, makes Indigenous people appreciate their *resguardos* even more. These are lands they cannot afford to give up, even if *resguardos* are increasingly unable to satisfy pressing everyday (material) needs, especially as a result of land appropriation by different powerful economic actors.

Overall, *resguardos* provide for less tangible elements of social reproduction such as a *sense of belonging*—which they cannot find elsewhere, even if the entire nuclear family has resettled and remains together. And although different motivations behind the creation of *resguardos* greatly contradicted Indigenous peoples’ worldview and traditions, as well as their concept of territory, *with time* these lands have come to represent an important source of emancipation and a tool to circumvent the more Western notions of property. Colombian anthropologist Laura Calle, who has dedicated her career to understanding indigenous cultures and ways of living across the eastern plains, is largely sympathetic with this proposition. She elaborates:

[…] we see that the legalization of the reservations was a demand of the Indigenous population from Andean indigenous organizations. Those people who did not live in reservations were trained on what this meant and also on the importance of having a council to be able to administer it as required by Colombian laws. The Sikuani incorporated the *resguardo* as a territorial model not because it was appropriate for their people or because it was based on their cultural particularities but because it was the clearest option in terms of the fight for the territory (Calle 2023:220–21).

During my fieldwork, I also visited the Indigenous *resguardos* of La Llanura and Campo Alegre y Ripialito in La Primavera, Vichada—from which various inhabitants of Unuma had migrated (see Fig. 1.2). My immediate feeling was far distant from that of a *resguardo* being an emancipatory tool. The almost seven-hour drive from the town centre made it an exhausting journey, despite the fact that I, and members of the Claretian organization, were travelling in the comfort of a rented powerful four-wheel drive van. Once in the *resguardo* we still needed to walk a considerable distance to collect water for personal hygiene and cooking, adding to the exhaustion. Indigenous repeatedly complained these same activities were becoming more difficult as a result of the restrictions imposed by private landowners and companies, many of whom partook in the recent land rush. And so, I initially left with the question of whether the struggle for land access in the form of *resguardos* was worthwhile at all. But as one activist and human rights defender once explained to me: ‘if we do not support indigenous’ struggles to preserve their *resguardo* lands, where else could they go?’. These seem to be the only available options.

Members from both *resguardos* I visited have repeatedly asked for the expansion of their territories before the Land Agency from around 2018, and have continuously claimed for improved land tenure security over the lands already certified as their own. Unfortunately, most petitions have fallen on deaf ears. It is impossible to ensure whether the situation would be different for the indigenous communities if these factors associated with land property change. However, based on the conversations I held, it is fair to say that if effective and real access by indigenous to their *resguardo* lands were guaranteed on the part of the state, many of those settled in Unuma today would not hesitate to return home. In the meantime, most continue to visit La Llanura and Campo Alegre as often as they can. After all, and reiterating a point made earlier, *resguardo* lands are important places that connect Indigenous peoples with their identity as a social group:

The land is our second mother, which sustains our life; There we were born, we grew. What we don’t want is for others to come and use the land and sell it, there can be no business with the land. If we sell the land, we cannot eat […]. For us, negotiating with the land is like selling our mother!

There in the *resguardo*, we had our *conucos* (bitter cassava, sweet cassava, chilli, cane, pineapple). We had everything: the river, there was plenty of fish, we didn’t need to buy, we worked the land by our hands […]. We are fighting to recover this territory to work the land because everything is produced from there, one can raise animals, plant crops, one can live. Without land, there is no life!

Our idea is to plow a few hectares in the savanna so that our peoples can sustain by themselves. We want to grow panela, rice, corn, have pigs, livestock; we want to recover this territory to work and to produce food (personal interviews, 06/2022).

In short, accounts from the indigenous migrant laborers in Puerto Gaitán suggest that it is neither one nor the other, i.e. *resguardos* lands or the lands of the informal urban settlement of Unuma, it is the two together that provide for the productive and social reproduction needs of the indigenous today. While at the informal indigenous settlement of town indigenous certainly alleviate pressing everyday needs, still other important elements of their reproduction as a collective are missed. As we saw, indigenous strive to remain connected to their ancestral lands and related natural resources, upon which they have typically based their subsistence. It is in this way that rural *resguardo* lands may be considered as one concrete expression of the use value of land in the context of the production-social reproduction matrix—even though they do no longer function in a day-to-day basis, as a result of land grabbing and dispossession, and the related increasing rate of indigenous migrating in search of wage work. More dedicated empirical research can help to further substantiate these propositions.

## Discussion and conclusion: land access in wider social reproduction’ terms and its implications for social justice struggles

The case of the indigenous migrant workers based in Puerto Gaitán is an example of the *continuing* relevance of land to people’s lives and livelihoods, despite of—or even because of—reallocation due to labor migration. As the previous two sections detailed, production and social reproduction take place both at the destination site of migrant work and at their rural home sites, often simultaneously, as it is also true with respect to other contexts (see Shah and Lerche 2020). Most notably, in these two areas, productive and reproductive needs are mediated by the ‘politics of land’ or “who gets what type, degree, and quality of access to which types of land, how, and for what purposes” (Borras et al. 2022:330). In this section, I discuss the implications of such interconnections between labour, production-social reproduction and land for social justice struggles.

The first implication is that efforts at strengthening land access, by state authorities and civil society organizations (e.g., The Claretians, for the purposes of our case) alike, must take into consideration a *wider* understanding of social reproduction. In the past, these efforts have been typically guided by a productivity rationale alone, in which land is allocated to grow food for self-consumption and/or to invest in commercial cultivation. Nevertheless, efforts must also reflect the broader elements that make up for social reproduction, many of which are less tangible and obvious—in comparison to the more material aspects of reproduction (e.g., provision of food, shelter, overall public services and care) (see Pattenden 2018). Key elements of this wider understanding of social reproduction are related with the communal, social and cultural bases attributed to land, which is equally fundamental to sustain (indigenous) peoples’ lives.

Both the material and the socio-cultural elements of social reproduction necessitate land for their realization. In this respect, Ossome (2021) notes,, the urgency of working towards the “decommodification of land”. This constitutes, in her view, “a shift away from the measurable, the quantifiable, the knowable, and thus the saleable”, in line with a “notion of land as uniquely valuable through the social entitlements it accords to people, and as priceless, and as mediating societal relations which exceed the market for it”. In the contemporary era, she stresses, the increasing commodification of land under ‘racial’ capitalism has dramatically “strips land off such symbolic, cultural significance” (2021:558). While acknowledging the complexities surrounding indigenous territories, it is possible to suggest that indigenous *resguardos* in Colombia are illustrative of the “symbolic significance” attributed to land.

The second implication, informed by the material and socio-cultural aspects of social reproduction, has to do with the increasing *multisitedness* of production and social reproduction and its effects upon land access. Inherently, such *multi-sited* character of the production-social reproduction nexus suggests the need to procure a wider access to land both in the *rural* areas, where the home sites are located, as well as in the *urban/peri-urban* destinations of migrant work. In this regard, Pattenden has recently put forward: “[…] given that most rural households make a living in both rural *and* urban locations, a primarily agrarian-focused approach to progressive politics is increasingly inadequate” (2023:17). This resonates strongly in the case of Colombia—albeit with some caveats, as explained in the below paragraphs.

As described above, although indigenous peoples across the eastern plains are still undoubtedly rooted to their rural *resguardo* lands, many of them are increasingly adding to the ranks of the ‘working people’—trying to make ends meet by engaging in multiple jobs outside their lands, many of which are non-agrarian, and increasingly too at peri-urban areas. Suppose, for example, that indigenous are provided with land tenure security over their *resguardos*, by which lands occupied by private landowners and companies are restituted to the communities through state law enforcement. Still, many challenges would have to be resolved before indigenous could achieve effective control of their lands in practice, in order to provide for a sustainable livelihood. But even if they manage to make real use of their *resguardo* lands in their inside, the overall social and material conditions in the surrounding area must be transformed as well (e.g. road infrastructure, health and social security services)—which is at the same a necessary, and a challenging and long-time endeavor. This is a time that indigenous peoples of Colombia, as elsewhere, do not have. This is also the primary reason why they were forced to migrate to the peri-urban area of Puerto Gaitán, in the first place.

Indeed, given the difficulties to access the means for production and for the social reproduction inside the *resguardos*, indigenous peoples in my research site must engage in a number of different activities both in the rural and peri-urban areas. Nonetheless, the reallocation of indigenous in peri-urban Puerto Gaitán cannot be paired with an outright detachment with their rural base (see Chambati 2023). It does not mean, either, that the focus for progressive politics in this case is now largely located in the urban rather than in the rural (Pattenden 2023). Arguably, the indigenous at Unuma might be seen, instead, as exemplifying what Borras (2023) has referred to as the “agrarian *and* non-agrarian rural” (original emphasis). This category results from combining another two, namely (i) the “agrarian” and the (ii) “non-agrarian rural”. He defines the first category: “social groups that remain primarily agrarian in character, whether farming (including swidden farmers), animal herding (including mobile pastoralists) or dwelling in forests, grasslands and marshlands (e.g., many Indigenous Peoples and many other farmers, fishers and landless agricultural labourers)”. The second category, he explains, “refers to people who live and work in rural societies (usually in small and medium rural towns) but are not engaged in any form of cultivation or pastoralism. They comprise an assortment of working classes in both the informal and formal sectors of the local economy” (2023:470–71). In this light, while rural-urban corridors are crucial an axis of political mobilization today, it does not make access to land in the rural areas, and the agrarian movements advocating for it, less important. Instead, it makes the struggles for land access, and the consequent mobilization of the working classes, more challenging than ever (Borras 2023:472; Borras and Franco 2023:49–53).

In the town of Puerto Gaitán, then, a broader access to land is central to indigenous’ subsistence, without it meaning that the land question of indigenous in the rural areas is less of a concern. Here I adhere to Calle’s (2024) call to deepen our understanding of indigenous territoriality, specifically in relation to their ancestral lands; and to recognize the historically specific forms of dispossession shaping the eastern plains—which provide valuable insights for understanding current demands for land recognition and the complexities surrounding the figure of *resguardo*. In what terms and for what purposes should struggles for better land access in Unuma be framed? This brings us to the third implication, necessarily linked with the above two. At Unuma, indigenous strive for material reproduction in a tiny piece of land where “there is no space to grow food or to have animals”—as indigenous I talked to repeatedly expressed. They struggle to satisfy their food needs at the lowest possible cost so that they can sell their labour power cheaply (Fraser 2016; Katz 2001) and remain competitive in the highly disputed labor market of Puerto Gaitán. As described earlier, the difficulties often faced by indigenous for commuting to the *resguardos* has meant, among others, that they have less access to their traditional foods. For the most part, instead, they must resort to the purchase of high-calorie foods and drinks that are usually the less expensive ones in the market—e.g., soda, potato chips. Therefore, in urban areas like those where Unuma is located, a better access to land is indispensable for indigenous migrant workers’ food provisioning—not just for the reproduction of labor, but essentially for their reproduction as people, just as it is central in their rural home areas. Ultimately, this complicated landscape of land, labor, production and social reproduction suggests the need to move from the often too ‘agrarian’ focus of the current debates on the agri-food system—and possible alternative pathways (e.g. food sovereignty) (see e.g. Borras et al. 2022:332; Pattenden 2023)—to look deeper into the complex intersections between social reproduction, labor migration and land access in the contemporary era. These intersections are increasingly rural-urban in character, in which the ‘rural’ continues to play an important role (Borras 2023), as illustrated by the socio-cultural significance attributed to rural indigenous lands (Calle 2024) and the struggles to retain access to them in the Colombian *Altillanura.*

## Footnotes

1 “Commodity and land rushes and regimes: Reshaping five spheres of global social life” (RRUSHES-5).

2 See also: Borras et al. (2022), Cousins (2022; 2018).

3 Access to land is also indispensable “for the establishment of a homestead” (Cousins et al. 2018:1079) and for the “structuring of social relations and identities” (2022), as well as for the construction of public service facilities (schools, hospitals) and recreational spaces (playgrounds, community centres); it enables access to other resources (water, forest), and it is also valued as a means for human beings’ realization on the whole (see Borras et al. 2022; Ojeda 2021).

4 This region is comprised by seven municipalities in two provinces of the eastern lowlands– Puerto Gaitán, Puerto López and Mapiripán in Meta, and the four municipalities integrating the neighboring province of Vichada (DNP 2014) (see Fig. 1). Of these all, Puerto Gaitán is the most dynamic one and strategically situated, to the point of it being resembled to a “natural paradise” (Gallo 2021; Ramírez 2012).

5 “We define land rush as denoting a chaotic, relatively short-lived, historical juncture marked by a sudden surge in demand for land, accompanied by an extremely speculative and competitive, often violent and convulsive transition from one set of rules on commodity and land politics to another. […]. Land rush encompasses various elements, namely, land enclosures, land grabs, land deals, land acquisitions, commodity booms– small, medium and large– and multiple actors (state, non-state, corporate and non-corporate), and has distinctive socio-political features, namely, wild speculations, hyperbolic claims, fantastic spectacles, a convulsive atmosphere”(Borras and Franco 2024:2).

6 As noted above, here Unuma refers to the name of the indigenous settlement in peri-urban Puerto Gaitán– and it is different from the usage of the term in the broader Sikuani tradition.

7 A closer look at the practices of collective work under Unuma by the indigenous at Puerto Gaitán merits further investigation and thus can be seen as one key area for future research.

8 Other recent publications by the author explore these same dynamics in relation to Afro-Colombian communities, particularly in the southwest Pacific region in Colombia and other rural settings. See, Vélez-Torres (2016; Vélez-Torres et al. 2019; Velez-Torres and Lugo-Vivas 2021).
